# From non-targeted to targeted GC–MS metabolomics strategy for identification of TCM preparations containing natural and artificial musk

**DOI:** 10.1186/s13020-022-00594-8

**Published:** 2022-04-01

**Authors:** Meng Ding, Jun-Li Fan, Dong-Fang Huang, Yue Jiang, Meng-Ning Li, Yu-Qing Zheng, Xiao-Ping Yang, Ping Li, Hua Yang

**Affiliations:** 1 State Key Laboratory of Natural Medicines, School of Traditional Chinese Pharmacy, China Pharmaceutical University, No. 24 Tongjia Lane, Nanjing, 210009 China; 2 Zhangzhou Pien Tze Huang Pharmaceutical Co., Ltd, Zhangzhou Fujian, 363000 China; 3 Fujian Provincial Key Laboratory of Pien Tze Huang Natural Medicine Research and Development, Zhangzhou Fujian, 363000 China

**Keywords:** Moschus, Androsterone, Prasterone, Muscone, Pien Tze Huang, Gas chromatography-mass spectrometrys, Metabolomics

## Abstract

**Background:**

*Moschus* is a rare and precious natural medicine. Due to the properties of resources scarcity and expensive price of natural musk, artificial musk has been developed as substitute materials in some prescriptions. Rapid and accurate identification of natural or artificial musk in complex traditional Chinese medicine (TCM) preparations is also a challenge.

**Method:**

A strategy from non-targeted to targeted gas chromatography-mass spectrometry (GC–MS) metabolomics was developed for discrimination of natural and artificial musk. Firstly, GC–MS-based non-targeted analysis combined with chemometrics was used to find the potential chemical markers to distinguish natural musk and artificial musk. Subsequently, targeted metabolomics was used to analyze musk in preparations with multiple reaction monitoring (MRM) mode by use gas chromatography coupled with triple quadrupole mass spectrometry (GC-QQQ MS).

**Results:**

Two chemical markers named prasterone and androsterone have been selected and could be detected in all Compound Pien Tze Huang preparations (CPZHs) containing artificial musk, while the CPZHs containing natural musk did not detect two markers with *S/N* (signal to noise ratio) less than 3.

**Conclusion:**

Our work provides an applicable approach to select the practical chemical markers for the assessment of musk in preparations to realize the traceability of musk in TCM and improve the quality control of musk-containing preparations.

**Supplementary Information:**

The online version contains supplementary material available at 10.1186/s13020-022-00594-8.

## Introduction

*Moschus* (natural musk), one of the most rare animal medicine in China with high price, is derived from the dried secretion in musk gland of mature male forest musk deer (*Moschus berezovskii*), alpine musk deer (*M. sifanicus*) or Siberian musk deer (*M. moschiferus*) [1, 2]. It has over 2000-year history of application in China for the treatment of coma, stroke, angina pectoris, chronic viral hepatitis and sprains. In recent decades, many efforts have been devoted to explore the chemical composition and pharmacological effects of natural musk. Natural musk is a complicated chemical system that mainly included macrocyclic ketones [3, 4], pyridine alkaloids [5], steroids [6, 7], peptides [8], proteins [9], fatty acids [10] and amino acids [8]. Among these ingredients, macrocyclic ketones and water-soluble peptides were the well-known active components and proved to possess prominent anti-inflammatory, neuro- and cardiovascular-protective effects [8, 11, 12]. For instance, muscone, a typical aromatic component of macrocyclic ketones, could regulate Drp1-dependent mitochondrial dynamics and thus ameliorate inflammatory responses and neuronal damage in a rat model of chronic cervical cord compression [13].

Because of its significant biological activities, natural musk has been utilized as an indispensable ingredient in a series of famous TCM preparations, such as Angong Niuhuang Pill, Pien Tze Huang and Liushen Pill. However, the population of musk deer has declined and become an endangered species under first rank protection in China due to overhunting and habitat loss [14]. For the sake of solving resource shortage of natural musk, artificial musk was thus developed in 1994 based on similarities in chemical, biological and physical properties of natural musk [15]. Currently, most musk-containing TCM preparations substituted natural musk with artificial musk. As a national secret recipe, the detailed components and their mixing proportion of artificial musk were not known. Only some predominant components such as muscone were well investigated [16]. Therefore, a rapid and reliable method to distinguish TCM preparations containing natural or artificial musk is essential for the assurance of safety and effectiveness.

Metabolomics-driven technological advances allow scientists to better understand the chemical constitution and comparison among different phenotypes [17]. The non-targeted metabolomics emphasized on the comprehensive analysis of all the detectable small molecules, and discovery of potential differential metabolites behind the specific phenomenon [18]. While the targeted metabolomics aims to quantitatively analyze a selected group of analytes to decipher the metabolic changes of interest or validate the chemical markers identified by using the untargeted metabolomics [19]. Combined the non-targeted and targeted metabolic profiling could thus contribute a deeper insight into discovering and evaluating the specific metabolites.

**Fig. 1:**
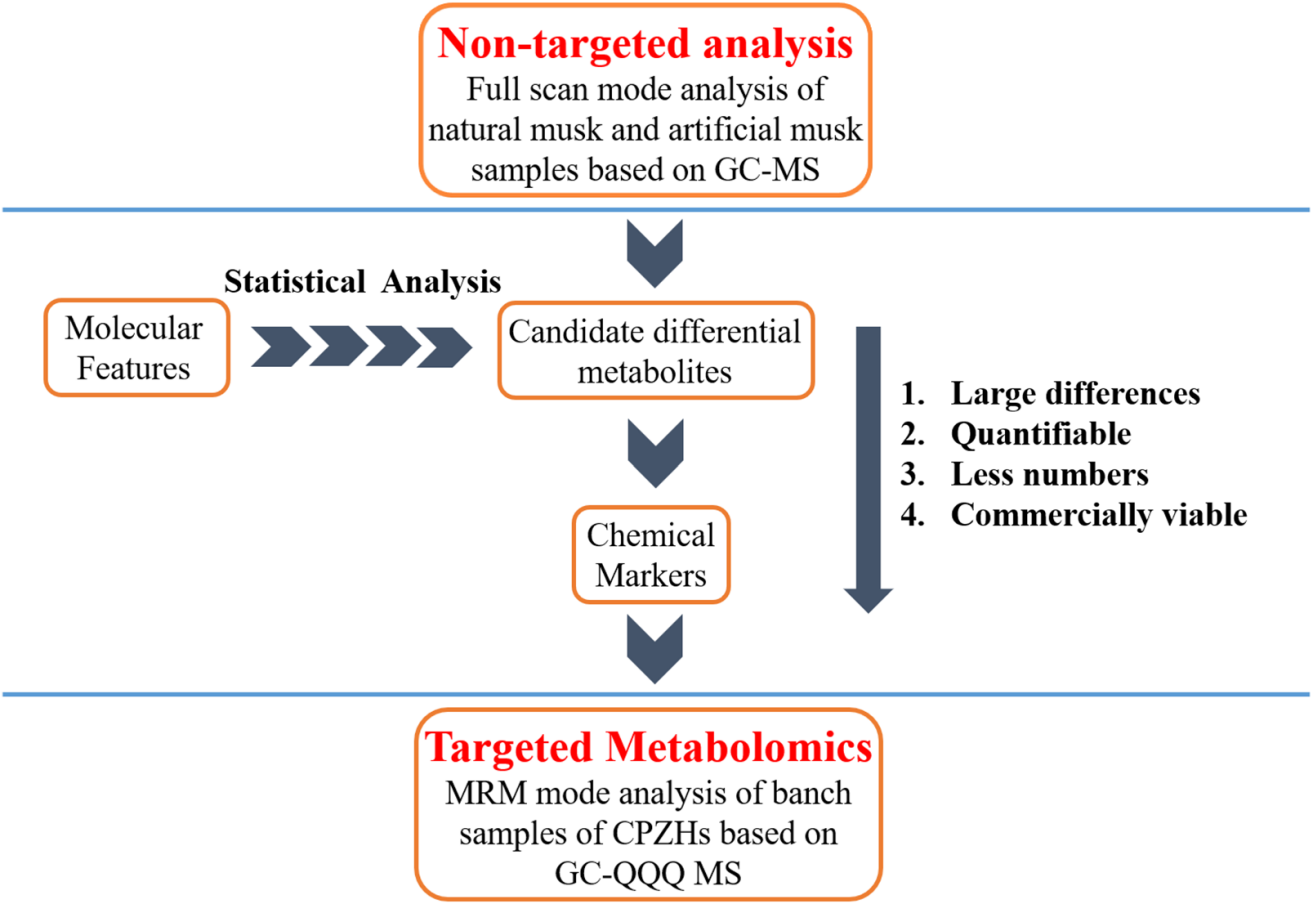
Frame of “Non-targeted to targeted GC–MS Metabolomics” analysis approach

Aim to accurate identification of TCM preparation containing natural or artificial musk, an integrated strategy from untargeted to targeted GC–MS metabolomic analysis was set up (Fig. 1). A comparative non-targeted analysis by GC–MS was for in-depth chemome comparisons between natural and artificial musk to obtain the optimal combination of chemical markers which meet the following four characteristics, including (1) large difference between the two groups; (2) quantifiable in at least one group of samples; (3) less numbers; (4) commercially viable, in other words, they can be easily obtained. For further identification of the musk sources in complex TCM preparation, the chemical markers were identified and applied to analyze three types of CPZHs by GC-QQQ MS based targeted metabolomic.

## Methods

### Materials and reagents

The 11 batches of natural musk, 10 batches of artificial musk and 3 types of CPZHs, including Compound-Pien-Tze-Huang-Buccal-Tablets (CPZHBT), Pien-Tze-Huang-Unguentum-Compositum (PZHUC) and Pien-Tze-Huang-Hemorrhoids-Ointment-Compositum (PZHHOC) were provided by Zhangzhou Pien Tze Huang Pharmaceutical Co., Ltd. CPZHBT is a tablet composed of Pien Tze Huang, Scrophulariae Radix and Glycyrrhizae Radix et Rhizoma, which is used to treat pharyngitis. PZHUC is an ointment composed of Pien Tze Huang powder and antivenom tablet which is used to treat dermatosis. PZHHOC is an ointment composed of Pien Tze Huang, Pearl powder and borneol which is used to treat hemorrhoids. Natural musk and artificial musk were authenticated by engineer Zhenzhen Jiang from Zhangzhou Pien Tze Huang Pharmaceutical Co., Ltd. All samples were stored dry at room temperature and protected from light. The detailed material information was shown in Additional file 1: Table S1 and Table S2.

Dichloromethane (CH_2_Cl_2_), ethanol (EtOH) and ethyl acetate (EtAc) of HPLC grade were purchased from Ruijingte Technology Co., Ltd. (Shenzhen, China). Reference substances of prasterone (LOT: 2113, > 98.0%) and androsterone (LOT: D1302001, > 98.0%) were purchased from Shidande Biotechnology Co., Ltd (Shanghai, China) and Yuanye Biotechnology Co., Ltd (Shanghai, China), respectively. Ultra-pure water (18 MΩ cm) was purified through a Millipore Milli-Q water purification system (Bedford, MA, USA).

### Chromatographic and mass spectrometric conditions

#### GC–MS analysis

The analyses of musk non-targeted metabolomics were performed on an Agilent 7890B gas chromatography coupling to Agilent 5977A mass spectrometry (Agilent Technologies, Santa Clara, CA, USA). HP-5 ms column (30 m × 0.25 mm, 0.25 μm) (Agilent Technologies) was used with helium as carrier gas (1 ml/min). 1 µl of sample was injected with a 5 min of solvent delay time and split ratio of 10:1. GC oven temperature was kept at 120 ℃ for 10 min and programmed to 190 ℃ at a rate of 20 ℃/min, and then programmed to 280 ℃ at a rate of 8 ℃/min, kept constant at 280 ℃ for 2 min. The injector, aux heaters, quadrupole and ion source temperature were set at 250 ℃, 280 ℃, 150 ℃ and 230 ℃, respectively. Mass spectra were recorded at 70 eV. The MS data were acquired in full scan mode from *m/z* 50-550.

The verification analysis of screened chemical markers was performed in the SIM mode using the target ion and confirmed by confirmative ions. The target ion of prasterone was *m/z* 288.2 and confirmative ions was *m/z* 91.1 and *m/z* 255.2 with retention time (RT) at 29.32 min. The target ion of androsteron was *m/z* 290.2 and confirmative ions was *m/z* 79.1 and *m/z* 107.1 with RT at 29.27 min.

#### GC-QQQ MS analysis

Identification of natural musk and artificial musk in CPZHs were performed on an Agilent 7890B gas chromatography coupling to Agilent 7000D triple quadrupole mass spectrometry (Agilent Technologies, Santa Clara, CA, USA). The collision cell gas, nitrogen flow was set at 1.5 ml/min and the quenching gas, helium flow at 2.25 ml/min. The column initial temperature was kept at 190 ℃. GC oven temperature was increased from 190 ℃ to 260 ℃ at a rate of 15 ℃/min and held for 5 min. The remaining chromatographic conditions are the same as the method described above. To develop an MRM method based on electron impact mass spectra, a unique precursor ion was selected followed at optimum collision energy (CE) to further fragment the precursor ion into product ions. The target ion of prasterone was *m/z* 288 and confirmative ions was *m/z* 270 with RT at 8.239 min at a CE of 10 eV. The target ion of androsteron was *m/z* 290 and confirmative ions was *m/z* 275 with RT at 8.296 min at a CE of 12 eV.

### Sample preparation

Each of natural musk and artificial musk dry powder was accurately weighed 50 mg and added into a 1 ml flask, added dichloromethane to the mark, immersed for 10 min, then ultrasound (40 kHz, 500 W) in ice-water bath for 30 min. After filtering with 0.22 μm filter membrane and centrifuging at 13,000 rpm for 10 min, 200 μl of the filtrate for GC–MS analysis of musk. 21 batches of sample solution were mixed equivalently as a quality control (QC) sample to test the reliability of the method. Take appropriate amounts of the prasterone and androsterone, add dichloromethane to make a reference substance solution containing 0.05 mg of each of the two reference substances per 1 ml.

The powder (1.5 g) of CPZHBT and ointment (0.2 g) of PZHUC and PZHHOC was weighted accurately and added into the 30 ml and 10 ml ethanol respectively for ultrasonically extraction at 40 kHz for 30 min with ice-water bath. The supernate was filtered through filter membrane (0.45 μm). Take half volume of the filtrate and nitrogen-blow it to dryness, add 0.5 ml of ethanol for secondary dissolution. The extract solution was centrifuged at 13,000 rpm for 10 min and preserved in 4 °C refrigerator before analysis. Take 200 μl of supernatant as the test solution for analysis.

Stock standard solution of prasterone and androsteron was prepared at the concentration of 1.24 mg/ml and 1.21 mg/ml in blank matrix solution of CPZHs and stored at 4 ℃ until use.

### Data processing

The GC–MS and GC-QQQ MS data acquisition and analysis was performed on MassHunter Workstation software (Agilent Technologies, Santa Clara, CA, USA). The compounds identification and comparison of reference substances through NIST17 database.

Import the raw data of 21 banches of musk samples and 4 QC samples into Agilent Unknows Analysis software (Agilent Technologies, Santa Clara, CA, USA) for deconvolution to screen differencial compounds. The results were exported as.cef files and imported into the MassProfiler Professional software (Agilent Technologies, 15.1, Santa Clara, CA, USA) for further peak alignment and filtering. The alignment parametars were set as follows: numbers of ions required set to 3, Match Factor set to 0.2, Delta MZ set to 0.2 and 0.1 min for retention time. The aligned data were filtered to retain only features with 50% presence in each group and subsequently screened by one-way analysis of variance (ANOVA) and fold change analysis (FCA). The final features were transformed to a binary logarithm (log2X, where X represents the peak area) in order to reduce the instrument error.

Principal component analysis (PCA) and orthogonal partial least-squares discrimination analysis (OPLS-DA) of musk metabolomics data was carried out by SIMCA-P Software 14.1 version (Umetrics AB, Umea, Sweden).

## Results and discussion

### Discovery of the chemical markers of natural and artificial musk by the non-targeted GC–MS metabolomics

Compound information of all musk samples was obtained in full scan mode. Comparing the total ion chromatograms (TICs) of the test solution of natural and artificial musk in Additional file 2: Figure S1, it was found that the peak intensities of multiple chromatographic peaks with the same retention time in natural and artificial musk have obvious differences. It preliminarily proved that natural musk and artificial musk were significantly different in chemical composition. In addition, the TIC overlay of 4 QC samples showed that the chromatograms basically overlapped, and the number of main compounds, response and retention time were basically the same (Additional file 2: Figure S2A. To further confirm the stability of methods and instruments, PCA was performed on 4 QC samples. The result showed that the projections of the 4 QC samples in the first principal component score plot are all within twice the standard deviation (SD) range (Additional file 2: Figure S2B), indicating that the data was reliable.

Untargeted metabolomic profiling followed by multivariate statistical analysis was applied to find differential metabolites based on relative contents. After alignment of peaks from 21 batches of musk, filtering the compounds using the algorithm of filtering by frequency, 160 potential differential metabolites were obtained with their *m/z*, RT and peak area (*Pa*). Subsequently, ANOVA and FCA were applied to figure out the features which had statistical difference (*p* value < 0.05 and FCA value ≥ 2.0) between the two groups. Based on this principle, 76 chemicals were picked out with significant differences. The resulting data was further exported for modelling analysis.

**Fig. 2:**
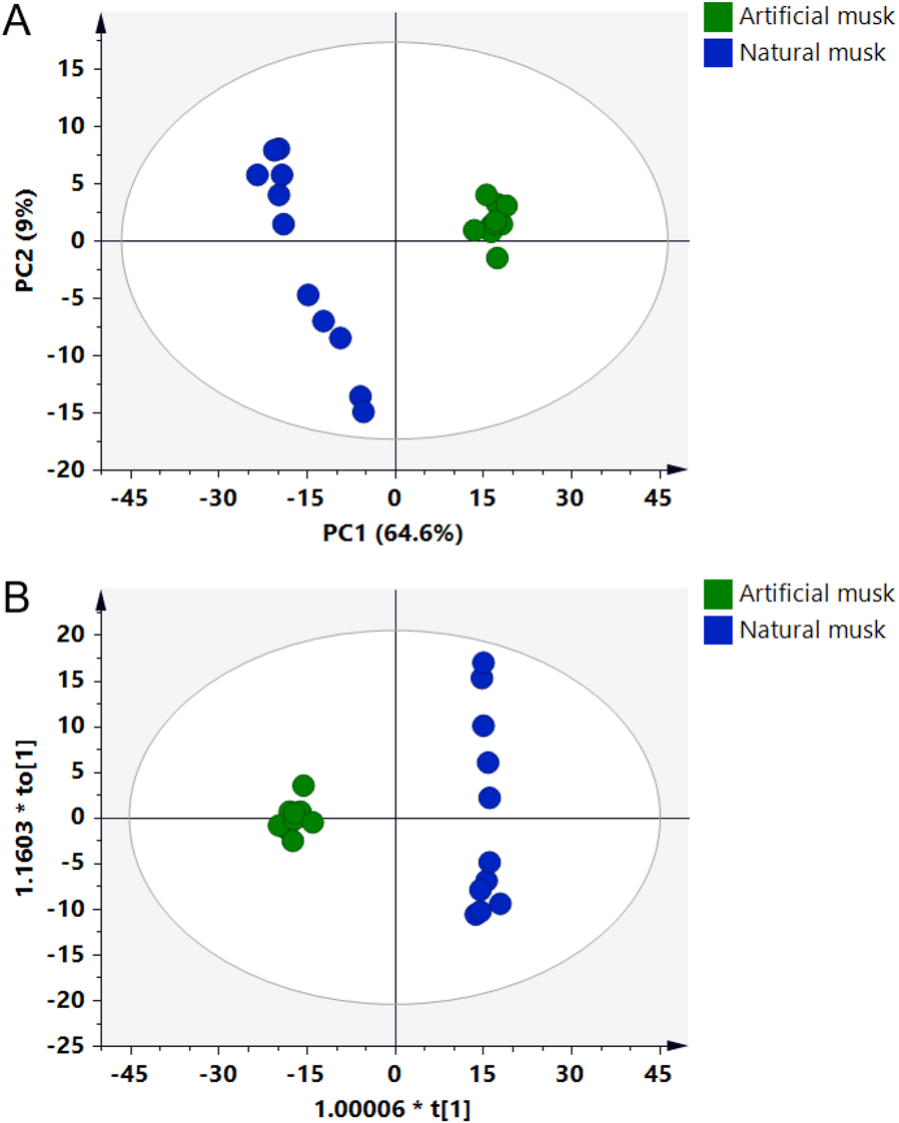
Principal component analysis (**A**) and orthogonal partial least-squares discrimination analysis (**B**) of natural musk (green) and artificial musk (blue)

PCA, an unsupervised pattern recognition method, could reveal the distribution and group relationship of samples through data dimensionality reduction [20, 21]. We utilized PCA to visualize the discrimination of natural musk and artificial musk on the the filtered 76 chemicals. The PCA score plot was classified into two zones according to natural and artificial musk (Fig. 2A). Supervised OPLS-DA model was subsequently employed to further divide the samples. Similar with the PCA results, the OPLS-DA scores plot (Fig. 2B) could also be readily classified into two zones, revealing a good classification and prediction ability of the model. Combined with the Variable Importance for the Projection (VIP), 36 differential metabolites were found from 76 metabolites with VIP value > 1, and these chemicals were considered potential differential components.

**Fig. 3:**
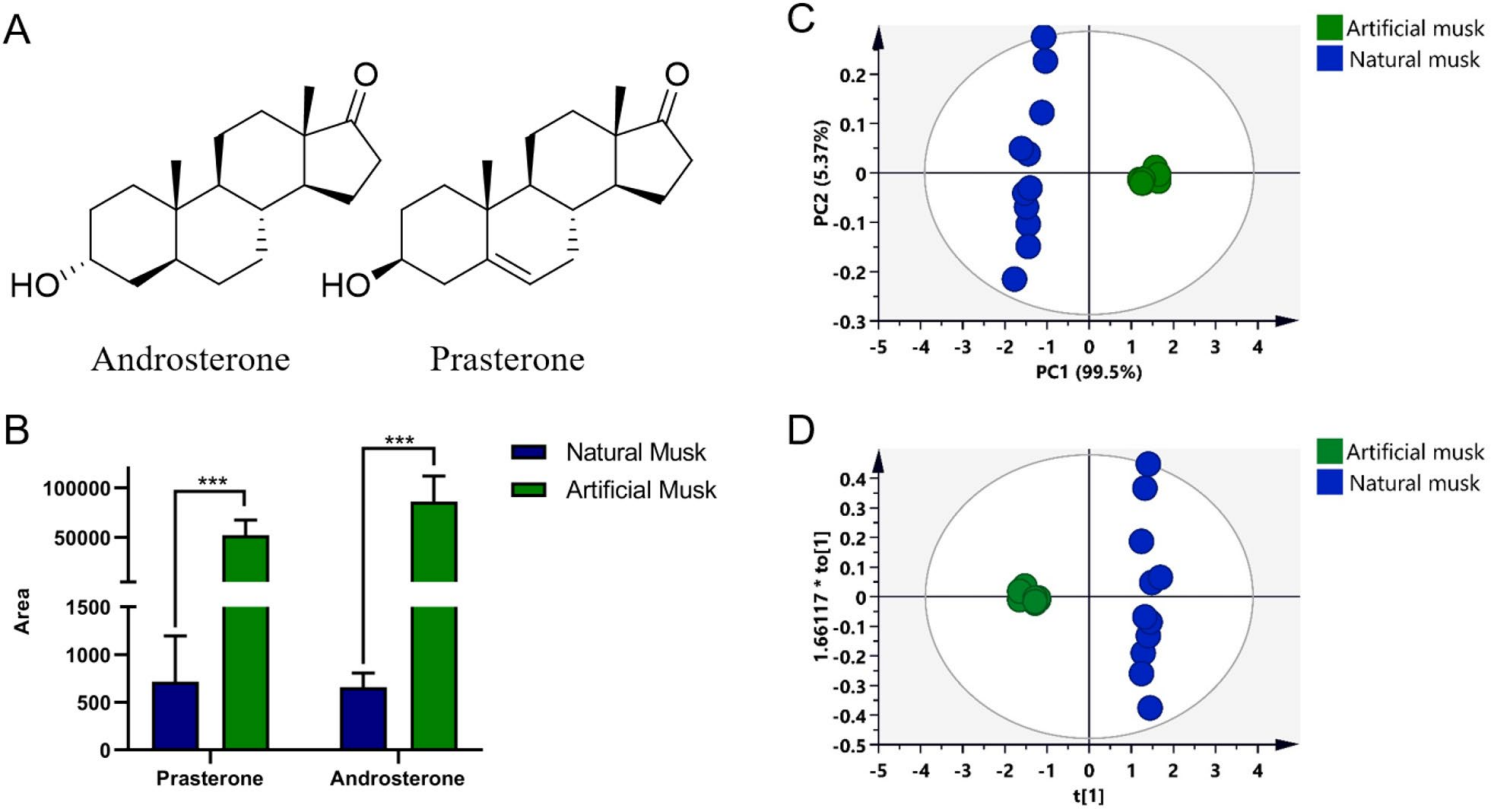
Chemical structures of the two chemical markers (**A**). Histogram (**B**) of the peak area in two markers. Principal component analysis (**C**) and orthogonal partial least-squares discrimination analysis (**D**) of prasterone and androstone in natural musk (green) and artificial musk (blue)

The 36 screened metabolites (VIP > 1) illustrated the different metabolic phenotypes of natural musk and artificial musk. Combined with NIST 17 library matching, standard comparison, and references, these potential differential components were identified (Table 1) and marked in the TIC of natural and artificial musk as shown in Additional file 2: Figure S1, including steroids, macroketones, fatty acids and their ester compounds [3, 10, 22]. In comparison to reference substances, two metabolites with the largest differences in the TICs and high VIP value between natural and artificial musk were accurately identified as prasterone and androsterone. Chemical structures of the two compounds are illustrated in Fig. 3.

**Table 1:** Identification of 36 differential ions with VIP > 1

No	Rt (min)	*m/z*	Identification	Formula	Match	VIP	Abundance^*a*^	
							AM	NM
1	6.09	85.10	2,6,10-trimethyltetradecane	C_17_H_36_	74.3	1.003	+ +	+ +
2	8.77	121.00	Ethylparaben	C_9_H_10_O_3_	96.2	1.799	+ + + +	–
3	9.03	223.20	P-ethoxybenzoic acid	C_9_H_10_O_3_	71.7	1.527	+ +	–
4	12.18	55.10	2-hexadecanol	C_16_H_34_O	72.7	1.454	–	+ +
5	12.98	149.10	Muscone	C_16_H_30_O	98.9	1.211	+ +	+ +
6	13.12	71.10	2-methylhexadecan-1-ol	C_17_H_36_O	76.0	1.445	+ + + +	+ + + +
7	14.26	174.10	Unknown	–	–	1.027	+	+ +
8	14.44	70.10	7-methyl-z-tetradecen-1-ol acetate	C_17_H_32_O_2_	78.1	1.373	+	–
9	15.47	59.10	Palmitic acid	C_16_H_32_O_2_	73.9	1.020	–	+ +
10	17.76	85.10	2-tetradecanol	C_14_H_30_O	76.0	1.144	+	–
11	18.9	98.10	L-ascorbic acid, Dihexadecanoate	C_38_H_68_O_8_	76.2	1.499	–	+ +
12	20.89	67.10	Linoleic acid ethyl ester	C_20_H_36_O_2_	84.7	1.551	+ +	–
13	21.51	59.10	Oleic acid	C_18_H_34_O_2_	72.7	1.173	+ +	+ + +
14	21.80	88.00	Arachidic acid	C_20_H_40_O_2_	76.8	1.527	+ +	+
15	23.21	91.00	Unknown	–	–	1.490	–	+ +
16	23.51	218.20	Unknown	–	–	1.004	+	–
17	23.93	130.00	Unknown	–	–	1.185	–	+ +
18	24.55	270.20	3-deoxyestradiol	C_18_H_24_O	73.4	1.431	+	+ +
19	26.38	71.10	3-deoxyestradiol	C_18_H_24_O	74.6	1.225	–	+ +
20	26.56	71.10	Unknown	–	–	1.178	+	+ +
21	28.53	129.10	Glycidyl oleate	C_21_H_38_O_3_	76.8	1.161	–	+ +
22	29.31	288.20	Prasterone*	C_19_H_28_O_2_	89.3	1.331	+ + + +	+ +
23	29.33	290.30	Androsterone*	C_19_H_30_O_2_	91.3	1.721	+ + + +	+ +
24	29.39	107.10	3-hydroxy-5β-androst-2-en-17-one	C_19_H_28_O_2_	84.3	1.124	+ + + +	+ +
25	29.99	99.10	Unknown	–	–	1.097	–	+ +
26	30.02	71.10	Unknown	–	–	1.099	–	+ + +
27	30.03	288.30	Androstanedione	C_19_H_28_O_2_	74.3	1.567	+ +	+
28	30.14	71.10	Unknown	–	–	1.555	–	+ +
29	30.56	117.00	Unknown	–	–	1.035	–	+ +
30	31.60	117.00	Unknown	–	–	1.027	+ +	+ + +
31	32.53	99.10	Unknown	–	–	1.269	–	+ + +
32	32.65	71.10	1,2-dipalmitoyl-sn-glycerol	C_35_H_68_O_5_	73.5	1.514	–	+ + +
33	33.06	69.10	Unknown	–	–	1.343	+	+ +
34	33.27	155.10	Unknown	–	–	1.068	–	+ +
35	33.51	127.10	Unknown	–	–	1.058	+	+ +
36	33.65	135.10	Unknown	–	–	1.173	+	+

^*a*^Abundance: peak abundances of EIC of differential ions in musk samples (AM and NM represented artificial musk and natural musk, respectively): “ − ”: No peaks were detected; “ + ”: 1000 ~ 10,000 counts; “ +  + ”: 10,000 ~ 100,000 counts; “ +  +  + ”: 100,000 ~ 1,000,000 counts; “ +  +  +  + ”: more than 1,000,000 counts. *: The identifications were confirmed with standard

Prasterone is an indirect precursor of estrogen, testosterone and other steroid hormones. Studies have shown [23] that it has a variety of pharmacological activities, such as anti-diabetic, anti-cancer, anti-allergic, obesity treatment and cardiovascular function, and is beneficial to autoimmune diseases. Androsterone is a metabolite of testosterone, has physiological activity on the central nervous system. Studies have demonstrated that androsterone has effect of anticonvulsant in both in vitro and in vivo experiments [24]. The physiological activities of the two chemical markers are closely related to the pharmacological effects of musk on the central nervous system, cardiovascular system, immune system and reproductive system. This work provides a reference for further research on the pharmacological mechanism of various activities of musk.

Previous studies [25, 26] have shown that natural musk contains a variety of androgen compounds, while artificial musk contains only a few androgen compounds, but the content is high. It is obvious that artificial musk uses a single component to replace a variety of complex similar compounds in natural musk, which is also the potential reason why androsterone and prasterone can be used as chemical marker to distinguish artificial musk from natural musk in our study.

### Specificity verification of chemical markers of natural and artificial musk

Selective ion monitoring (SIM) mode was performed on each batch of musk by using GC–MS, the chromatograms and *Pa* were recorded. The *Pa* histograms of the two markers in natural and artificial musk showed the content of prasterone and androsterone in artificial musk are significantly higher than those in natural musk (Fig. 3B). Import the normalized *Pa* into SIMCA P for PCA (Fig. 3C) and OPLS-DA (Fig. 3D), natural and artificial musk were clearly distinguished, indicating that the two differential compounds could distinguish natural and artificial musk. As is shown, 10 batches of artificial musk are closely clustered together, and 11 batches of natural musk are scattered on the other side. It indicated that the contents of prasterone and androsterone are relatively stable in artificial musk, but dynamic in different batches of natural musk. This could be caused by the wide range of natural musk sources and diverse production areas. Previous studies have reported that the total androgen in natural musk varies greatly in different regions, and the types and contents of steroids in different varieties of natural musk were diverse [4, 27].

### Identification of CPZHs containing natural and artificial musk by GC-QQQ MS based targeted metabolomics

#### Targeted profile of prasterone and androsterone

Take the reference solution of prasterone and androsterone in CPZHs (blank matrix), test solution of three CPZHs (CPZHBT (natural musk), CPZHBT (artificial musk), CPZHBT (blank matrix), PZHUC (natural musk), PZHUC (artificial musk), PZHUC (blank matrix), PZHHOC (natural musk), PZHHOC (artificial musk), PZHHOC (blank matrix) for the methodological specificity verification.

**Fig. 4:**
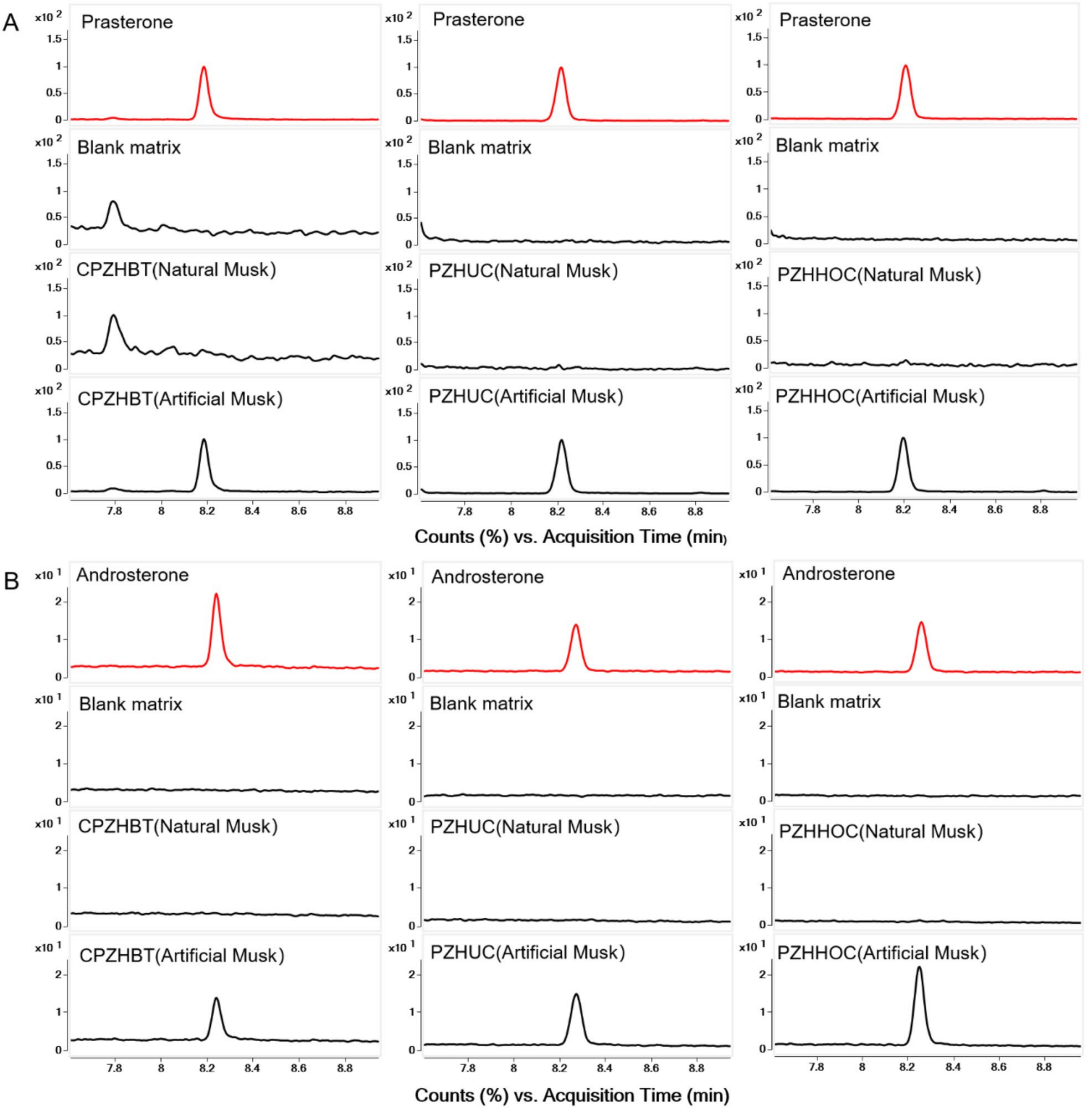
Specific identification of prasterone (**A**) and androsterone (**B**) in compound Pien Tze Huang Buccal tablets, Pien Tze Huang Unguentum Compositum and Pien Tze Huang Hemorrhoids Ointment Compositum

As shown in Fig. 4, The CPZHs (blank matrix) have no interference in the detection of the markers, the CPZHs (artificial musk) showed chromatographic peaks consistent with the RT of prasterone and androsterone (*S/N* > 3), while CPZHs (natural musk) were not detected prasterone and androsterone (*S/N* < 3). The *Pa* and *S/N* are shown in Additional file 1: Table S3. It demonstrated that prasterone and androsterone can be used as specific components to identify artificial musk in CPZHs.

The limits of detection (LOD) of prasterone in CPZHBT, PZHUC and PZHHOC was 8.93 ng/ml, 8.93 ng/ml and 14.88 ng/ml respectively. The LOD of androsterone was 34.57 ng/ml, 43.21 ng/ml and 43.21 ng/ml, respectively. Take the appropriate concentration of the reference solution for precision inspection. The relative standard deviations (RSDs) (n = 6) of RT and *Pa* were less than 0.07% and 2.51%, respectively (Additional file 1: Table S4).

To confirm the extracted repeatability, six independent test solutions for each preparation were prepared. All CPZHs (artificial musk) can detect prasterone and androsterone in the test solutions (*S/N* > 3). The RSDs of RT and *Pa* demonstrated that extraction repeatability was satisfactory, which were less than 0.04% and 7.82% (n = 6) (Additional file 1: Table S5). Prasterone and androsterone were not detected in all CPZHs (natural musk) (*S/N* < 3).

Prepare test solutions of CPZHs (natural musk) and CPZHs (artificial musk), and analyze the samples at 0 h, 4 h, 8 h, 12 h, 16 h, and 24 h after preparation for stability investigation. The RSDs of RT and *Pa* were less than 0.08% and 5.41% in CPZHs (artificial musk), proved that the sample is stable within 24 h. Neither compound was detected in CPZHs (natural musk) (*S/N* < 3) (Additional file 1: Table S5).

#### Real sample analysis

The extraction conditions of sample of CPZHs were investigated using the *Pa* of the MRM chromatographic peaks of two differential compounds as the inspection index. Dichloromethane, ethanol and ethyl acetate were compared as extraction solvents. Volume of extraction solvent was also investigated. It turned out that 30 ml ethanol has the highest extraction efficiency for CPZHBT and 10 ml ethanol has the highest extraction efficiency for PZHUC and PZHHOC. The investigation on extraction conditions of CPZHs are illustrated in Additional file 2: Figure S3.

**Fig. 5:**
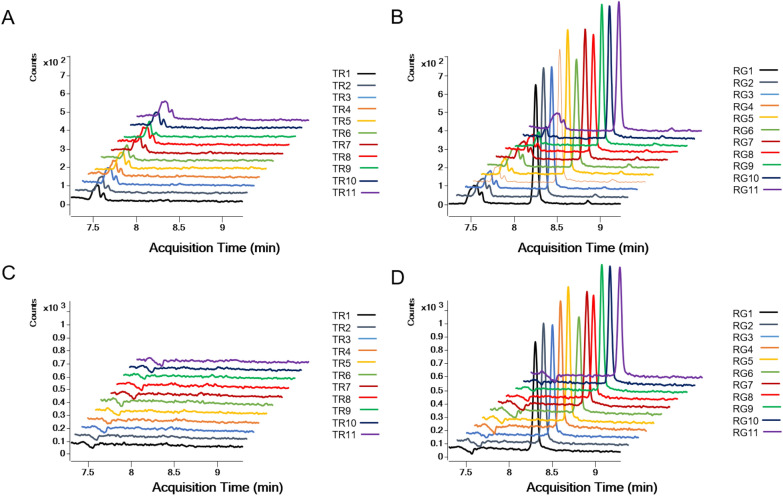
Sample determination of Pien Tze Huang Hemorrhoids Ointment Compositum. (**A**) Prasterone in samples of Pien Tze Huang Hemorrhoids Ointment Compositum. (Natural Musk); (**B**) Prasterone in samples of Pien Tze Huang Hemorrhoids Ointment Compositum. (Artificial Musk); (**C**) Androsterone in samples of Pien Tze Huang Hemorrhoids Ointment Compositum. (Natural Musk); (**D**) Androsterone in samples of Pien Tze Huang Hemorrhoids Ointment Compositum. (Artificial Musk)

Take the PZHHOC as an example, analyze the test solutions of 11 batches of PZHHOC (natural musk), 11 batches of PZHHOC (artificial musk) in MRM mode for sample determination. Record the MRM chromatograms of each batch of samples (Fig. 5) and the *Pa* and *S/N* of two markers in CPZHs were shown in Table 2. PZHHOC (artificial musk) showed chromatographic peaks (*S/N* > 3) consistent with the Rt of prasterone and androsterone reference substances, while neither compound was detected in the PZHHOC (natural musk) (*S/N* < 3). It indicated that prasterone and androsterone can specifically identify artificial musk in PZHHOC. Similar results appeared in the sample determination of CPZHBT and PZHUC as show in Additional file 2: Figure S5 and S6, respectively. The results of sample determination showed that the two chemical markers obtained based on metabolomics screening can be successfully applied to the identification of artificial musk in CPZHs.

**Table 2:** Peak area and signal to noise ratio (*S/N*) of prasterone and androstenone in three compound Pien Tze Huang preparations

	Compound Pien Tze Huang Buccal tablets					Pien Tze Hung Unguentum compositum					Pien Tze Huang Hemorrhoids ointment				
	Sample	Prasterone		Androsterone		Sample	Prasterone		Androsterone		Sample	Prasterone		Androsterone	
		Area	*S/N*	Area	*S/N*		Area	*S/N*	Area	*S/N*		Area	*S/N*	Area	*S/N*
Natural musk	N01	-	-	-	-	N12	-	-	-	-	N23	-	-	-	-
	N02	-	-	-	-	N13	-	-	-	-	N24	-	-	-	-
	N03	-	-	-	-	N14	-	-	-	-	N25	-	-	-	-
	N04	-	-	-	-	N15	-	-	-	-	N26	-	-	-	-
	N05	-	-	-	-	N16	-	-	-	-	N27	-	-	-	-
	N06	-	-	-	-	N17	-	-	-	-	N28	-	-	-	-
	N07	-	-	-	-	N18	-	-	-	-	N29	-	-	-	-
	N08	-	-	-	-	N19	-	-	-	-	N30	-	-	-	-
	N09	-	-	-	-	N20	-	-	-	-	N31	-	-	-	-
	N10	-	-	-	-	N21	-	-	-	-	N32	-	-	-	-
	N11	-	-	-	-	N22	-	-	-	-	N33	-	-	-	-
Artificial musk	A01	1472	136.50	1500	50.82	A12	1533	152.26	1960	74.87	A23	2090	166.23	2877	91.13
	A02	1680	156.97	1675	60.11	A13	2165	198.12	2660	82.09	A24	2277	132.71	2934	75.52
	A03	1540	170.86	1394	39.41	A14	2320	138.08	3127	65.03	A25	2002	208.39	2608	92.75
	A04	1195	96.35	1163	50.42	A15	1803	258.38	2352	80.36	A26	2238	203.79	3019	67.11
	A05	1054	135.87	989	30.34	A16	1702	293.47	2178	96.78	A27	2332	256.32	3080	100.51
	A06	1773	136.72	1748	84.92	A17	2103	178.02	2739	70.47	A28	1930	133.50	2590	104.73
	A07	1892	118.81	1840	62.96	A18	2242	135.05	2929	91.34	A29	2254	162.98	3007	53.77
	A08	1541	169.84	1558	50.01	A19	1960	192.82	2415	61.85	A30	1901	95.81	2464	92.71
	A09	1636	259.67	1711	43.36	A20	2142	255.93	2777	99.82	A31	2184	139.57	2902	170.90
	A10	1463	156.69	1562	68.37	A21	2007	141.00	2565	118.15	A32	2111	179.70	2746	117.43
	A11	2246	80.99	2392	64.08	A22	1542	216.27	1869	76.45	A33	2289	137.88	2946	69.23

“-”: no peaks were detected (*S/N* < 3)

## Conclusion

In this study, an integration strategy from non-targeted to targeted GC–MS metabolomics was aiming to find chemical markers to identify artificial musk in TCM preparations characteristically. Prasterone and androsterone are screened out and successfully applied to specific identification of artificial musk in CPZHs, providing a reference to solve the quality problem that artificial musk mixed or misused in natural musk preparations. The quality control of natural musk in the preparation was improved. Two markers we found are also closely related to the pharmacological effects of musk, which might present the evidence for further research on origin identification, pharmacological effect, and quality control of musk and musk-containing preparations. Previous reported biological studies of two chemical markers have shown their potential correlation with the pharmacological effects of musk. Our research also provides some evidence that artificial musk can replace natural musk in some preparations.

## Supplementary Information

**Additional file 1:** **Table S1.** Sample information of natural musk and artificial musk. **Table S2.** Sample information of compound Pian Tze Huang preparations. **Table S3.** Specificity of the compound Pien Tze Huang preparations. **Table S4.** Precision of the compound Pien Tze Huang preparations (n = 6). **Table S5.** Repeatability and stability of the compound Pien Tze Huang preparations.

**Additional file 2:** **Figure S1.** Typical total ion chromatograph of natural musk and artificial musk. (*: Two chemical markers; 1-36 are listed in Table 1). **Figure S2.** Overlap of TIC (A) and PCA (B) of four quality control samples. Figure S3. Investigation on extraction conditions of CPZHBT (A), PZHUCC (B) and PZHHOC (C). **Figure S4.** Sample determination of CPZHBT. (A) Prasterone in samples of CPZHBT (Natural Musk); (B) Prasterone in samples of CPZHBT (Artificial Musk); (C) Androsterone in samples of CPZHBT (Natural Musk); (D) Androsterone in samples of CPZHBT (Artificial Musk). **Figure S5.** Sample determination of PZHUC. (A) Prasterone in samples of PZHUC (Natural Musk); (B) Prasterone in samples of PZHUC (Artificial Musk); (C) Androsterone in samples of PZHUC (Natural Musk); (D) Androsterone in samples of PZHUC (Artificial Musk).

## Data Availability

The datasets used and/or analysed during the current study are available from the corresponding author on reasonable request.

## Abbreviations

TCM: Traditional Chinese medicine
GC–MS: Gas chromatography-mass spectrometry
MRM: Multiple reaction monitoring
GC-QQQ MS: Gas chromatography coupled with triple quadrupole mass spectrometry
CPZHBT: Compound-Pien-Tze-Huang-Buccal-Tablets
PZHUC: Pien-Tze-Huang-Unguentum-Compositum
PZHHOC: Pien-Tze-Huang-Hemorrhoids-Ointment-Compositum
SIM: Selected ion monitoring
QC: Quality control
ANOVA: One-way analysis of variance
FCA: Fold change analysis
PCA: Principal component analysis
OPLS-DA: Orthogonal partial least-squares discrimination analysis
TICs: Total ion chromatograms
RT: Retention time
*Pa*: Peak area
VIP: Variable importance for the projection
