# Supplementary material for: From non-targeted to targeted GC–MS metabolomics strategy for identification of TCM preparations containing natural and artificial musk
Source: Chin Med. 2022 Apr 1;17:41. doi: 10.1186/s13020-022-00594-8 (PMC8974109; doi:10.1186/s13020-022-00594-8)
Supplement: Supplementary file 2 — Additional file 2: Figure S1. Typical total ion chromatograph of natural musk and artificial musk. (*: Two chemical markers; 1-36 are listed in Table 1). Figure S2. Overlap of TIC (A) and PCA (B) of four quality control samples. Figure S3. Investigation on extraction conditions of CPZHBT (A), PZHUCC (B) and PZHHOC (C). Figure S4. Sample determination of CPZHBT. (A) Prasterone in samples of CPZHBT (Natural Musk); (B) Prasterone in samples of CPZHBT (Artificial Musk); (C) Androsterone in samples of CPZHBT (Natural Musk); (D) Androsterone in samples of CPZHBT (Artificial Musk). Figure S5. Sample determination of PZHUC. (A) Prasterone in samples of PZHUC (Natural Musk); (B) Prasterone in samples of PZHUC (Artificial Musk); (C) Androsterone in samples of PZHUC (Natural Musk); (D) Androsterone in samples of PZHUC (Artificial Musk). [file 13020_2022_594_MOESM2_ESM.docx]

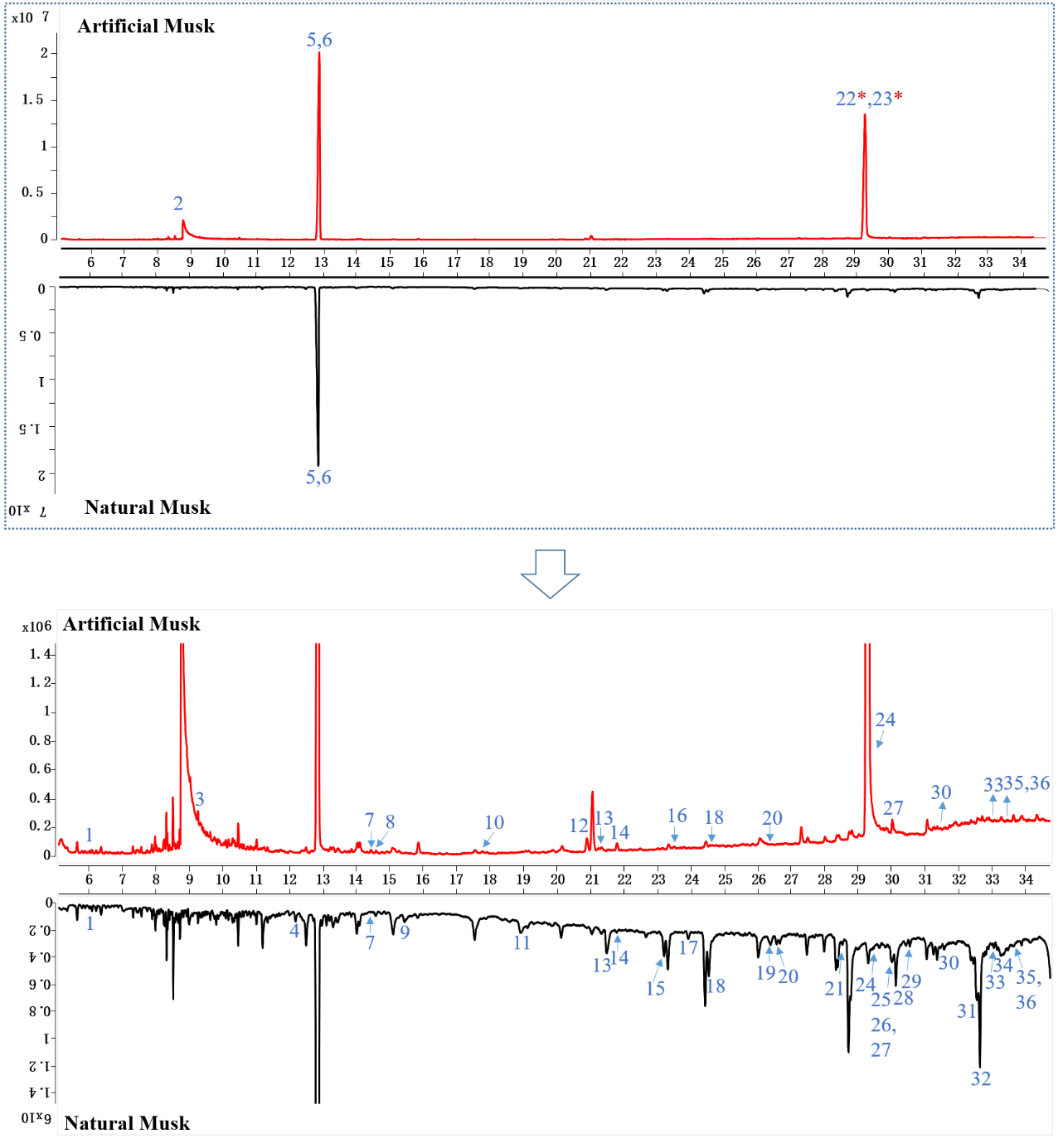


Figure S1. Typical total ion chromatograph of natural musk and artificial musk. (*: Two chemical markers; 1-36 are listed in Table 1).


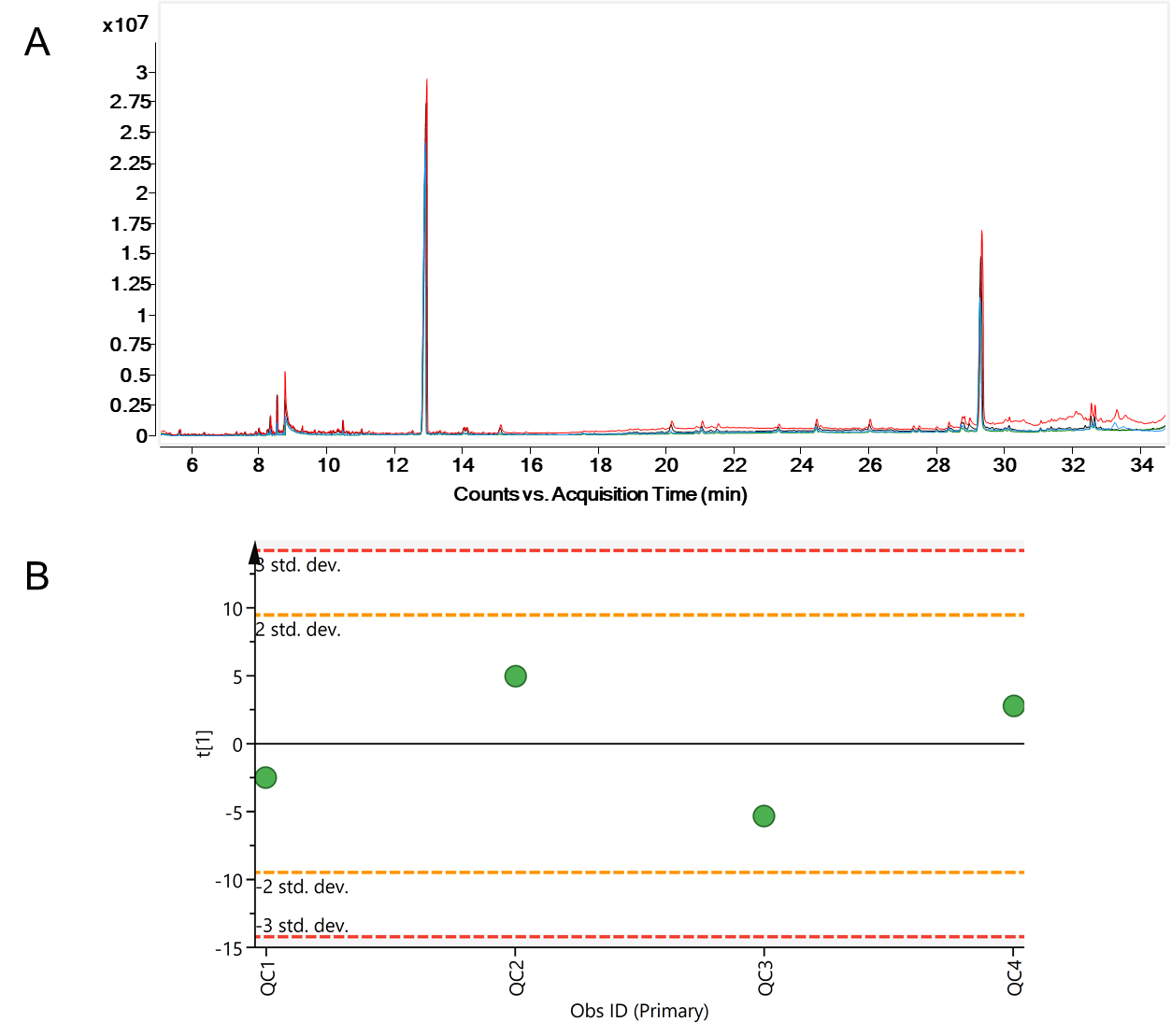


**Figure S2** Overlap of TIC (A) and PCA (B) of four quality control samples.


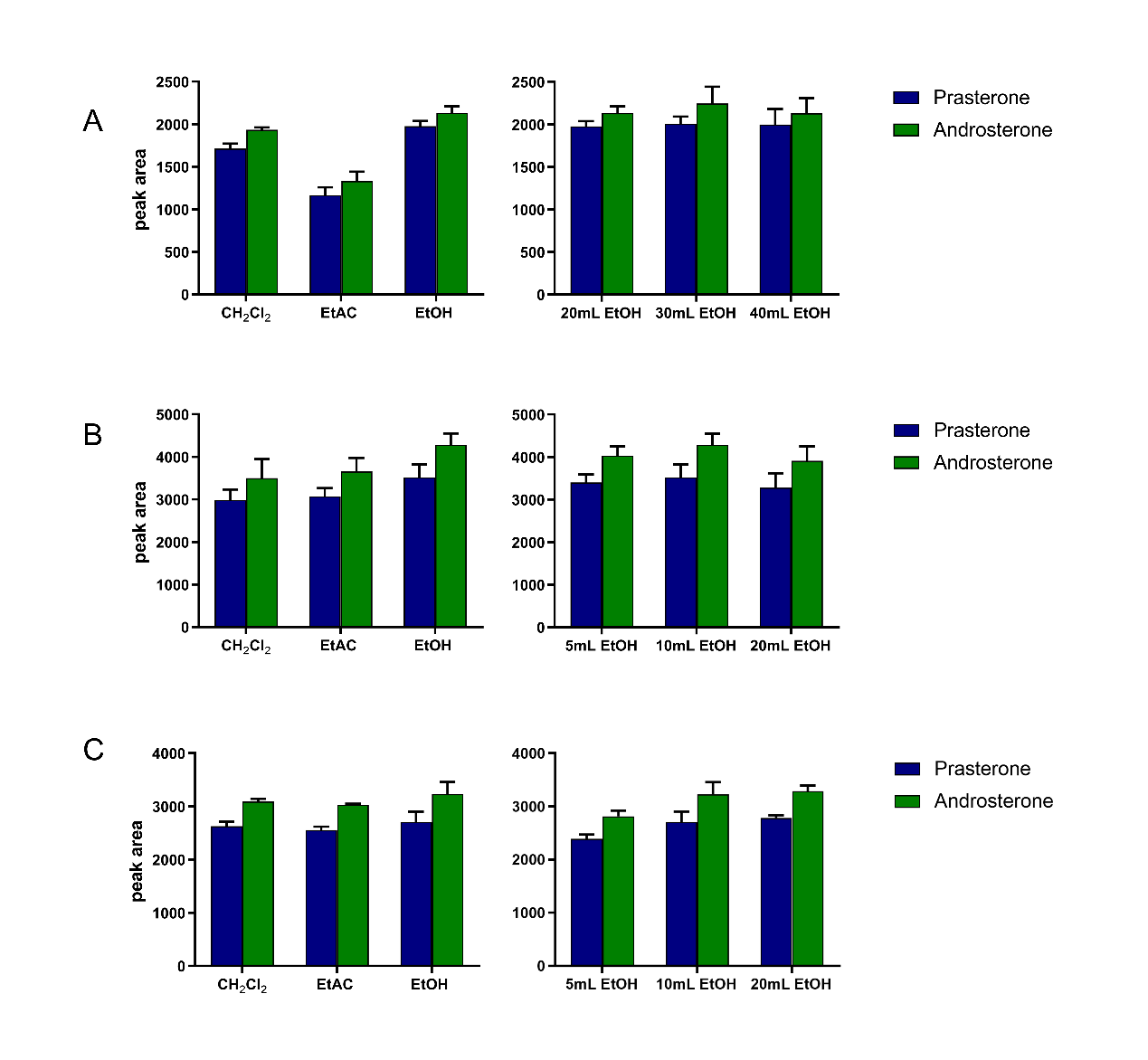


**Figure S3** Investigation on extraction conditions of CPZHBT (A), PZHUCC (B) and PZHHOC (C).


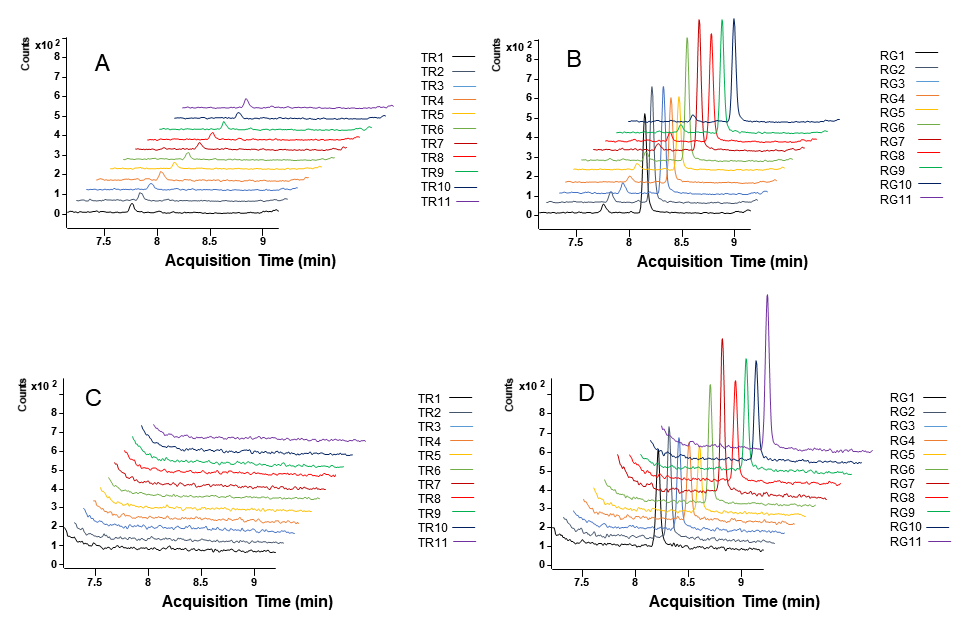


**Figure S4** Sample determination of CPZHBT. (A) Prasterone in samples of CPZHBT (Natural Musk); (B) Prasterone in samples of CPZHBT (Artificial Musk); (C) Androsterone in samples of CPZHBT (Natural Musk); (D) Androsterone in samples of CPZHBT (Artificial Musk)

**
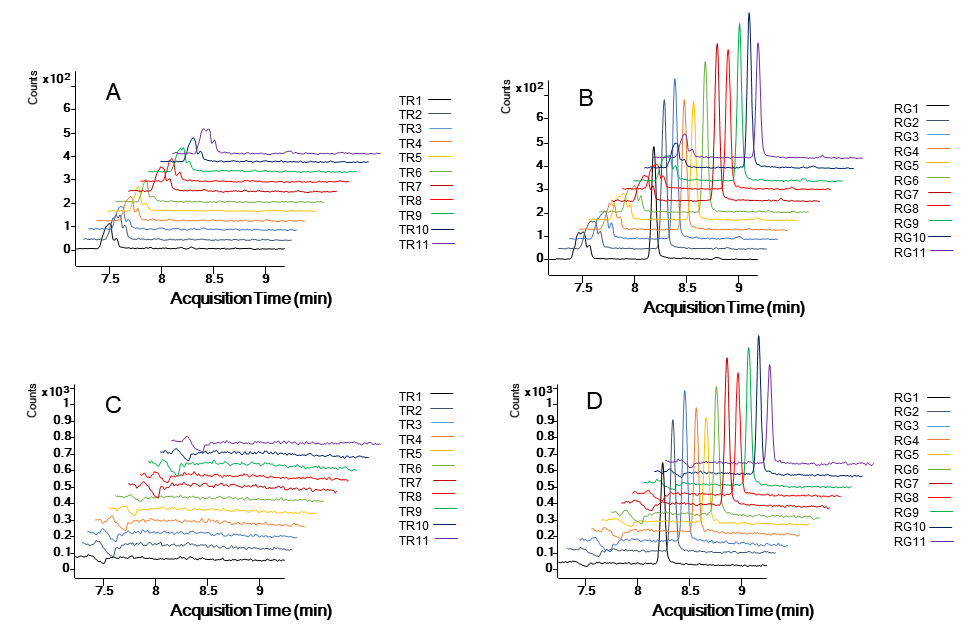
**

**Figure S5**. Sample determination of PZHUC. (A) Prasterone in samples of PZHUC (Natural Musk); (B) Prasterone in samples of PZHUC (Artificial Musk); (C) Androsterone in samples of PZHUC (Natural Musk); (D) Androsterone in samples of PZHUC (Artificial Musk)
